# Are conventional microbiological diagnostics sufficiently expedient in the era of rapid diagnostics? Evaluation of conventional microbiological diagnostics of orthopedic implant-associated infections (OIAI)

**DOI:** 10.1080/17453674.2020.1844499

**Published:** 2020-11-10

**Authors:** Hege Vangstein Aamot, J Christopher Noone, Inge Skråmm, Truls M Leegaard

**Affiliations:** a Department of Microbiology and Infection Control, Akershus University Hospital, Lørenskog;; b Faculty of Medicine, University of Oslo, Oslo;; c Orthopedic clinic, Akershus University Hospital, Lørenskog;; d Institute of Clinical Medicine, Faculty of Medicine, University of Oslo, Oslo, Norway

## Abstract

Background and purpose — In a time when rapid diagnostics are increasingly sought, conventional procedures for detection of microbes causing orthopedic implant-associated infections (OIAI) seem extensive and time-consuming, but how extensive are they? We assessed time to (a) pathogen identification, (b) antibiotic susceptibility patterns, and (c) targeted antibiotic treatment using conventional microbiological diagnostics of OIAI in a consecutive series of patients.

Patients and methods — Consecutive patients aged ≥18 years undergoing first revision surgery for acute OIAI, including prosthetic joints, fracture, and osteotomy implants, in 2017–2018 at Akershus University Hospital (Ahus), Norway were included. Information regarding microbiological diagnostics and clinical data was collected retrospectively from the hospital’s diagnostic and clinical databases.

Results — 123 patients fulfilled the inclusion criteria. Median time to pathogen identification was 2.5 days and to antibiotic treatment recommendations was 3.5 days. The most common pathogens were *S. aureu*s (52%) and *S. epidermidis* (15%). Cultures were inconclusive in 11% of the patients. Of the 109 patients with culture-positive results, antibiotic treatment was changed in 66 (61%) patients within a median of 4 days (0–24) after the recommendation was given.

Interpretation — Conventional microbiological diagnostics of OIAI is time-consuming, taking days of culturing. Same-day diagnostics would vastly improve treatment efficacy, but is dependent on rapid implementation by clinicians of the treatment recommendations given by the microbiologist.

The majority of orthopedic procedures include the use of implants, which increase the risk of infection due to the reduced number of bacteria needed to establish an infection (Zimmerli et al. 1982). Orthopedic implant-associated infections (OIAI) are infrequent per se, with an overall surgical site infection rate following implant surgery of 3% (Skråmm et al. 2012). However, the number of patients undergoing orthopedic implant surgery is high and increasing (Norwegian National Advisory Unit on Arthroplasty and Hip Fractures 2020).

A microbiological diagnosis is vital for providing the best treatment, with regards to both surgical options and providing targeted and narrow-spectrum antimicrobial therapy (Beam and Osmon 2018). Today’s conventional diagnostics include microbiological culturing of 5 biopsies from each infected patient on several different media for at least 5 days dependent on growing and dividing bacteria (Bergh et al. 2011, Osmon et al. 2013). More rapid diagnostic tools are being developed, but with varying degrees of sensitivity and specificity (Bonanzinga et al. 2017, Jun and Jianghua 2018, Aamot et al. 2019).

We assessed time to (a) pathogen identification, (b) antibiotic susceptibility patterns, and (c) targeted antibiotic treatment using conventional microbiological diagnostics of OIAI in a consecutive series of patients.

## Patients and methods

This retrospective cohort study included all patients aged ≥ 18 years operated for acute OIAI (including prosthetic joint infections, fracture implants, and osteotomy implants) undergoing first revision surgery in 2017–2018 at Akershus University Hospital (Ahus), Norway. Ahus is Norway’s largest acute care hospital serving > 10% of the Norwegian population (5.4 million inhabitants) and performs ∼4,000 orthopedic implant surgeries annually. The microbiology laboratory is situated in the center of the hospital with short, indoor transportation of patient samples from the operating theatre. In the study period, laboratory opening hours were 07:30–16:30 on weekdays and 07:30–15:00 on weekends with an extension from November 2018 to 07:30–21:00 on weekdays.

The criteria for an OIAI were based on the Modified Musculoskeletal Infection Society (MSIS) criteria described by Parvizi et al. (2011).

Conventional culturing was performed by homogenizing up to 5 tissue samples individually with mortar and pistil in heart infusion broth (HIB) in a type 2 microbiological safety cabinet with subsequent seeding as previous described (Aamot et al. 2019). Incubation was terminated after 5 days following consensus (Parvizi et al. 2013) unless slow growing bacteria, such as *Cutibacterium acnes*, were suspected to be clinically relevant. The incubation period was prolonged to 14 days in such cases. Cultivation results have previously been published in 13 patients (Aamot et al. 2019).

Microbe identification was performed by matrix-assisted laser desorption ionization time of flight (MALDI-TOF) using MALDI-TOF MS Biotyper (Bruker Daltonik GmbH, Bremen, Germany, MBT 6903 MSP Library, MBT Compass v4.1.70.1, Compass for flexControl v3.4). Antibiotic susceptibility testing was performed according to the guideline from the European Committee on Antimicrobial Susceptibility Testing EUCAST (EUCAST 2017a) and EUCAST breakpoints were utilized to categorize the isolate as sensitive (S), intermediate (I) (now susceptible, increased exposure), or resistant (R) (EUCAST 2017b).

Information regarding microbiological diagnostics and clinical data was collected retrospectively from the hospital’s diagnostic and clinical databases. Time to results were defined as the time from biopsy to pathogen identification, the time to antibiotic treatment advice and the time to completed analyses including anaerobic cultivation. Confirmed infection, microbiologically, was defined as identification of the same microbe in 2 or more patient samples, whereas unconfirmed infection was defined as identifying a microbe in fewer than 2 patient samples (Parvizi et al. 2018).

Empirical treatment was based on national guidelines (Norwegian Directorate of Health, 2020) and distributed intraoperatively after biopsy. For prosthetic joint infections (PJI), the empirical treatment was vancomycin, ciproxin, and/or dicloxacillin. For other implant infections, the empirical treatment was penicillinase-resistant penicillin. 11 patients received non-empirical, targeted treatment prior to surgery due to previously diagnosed bloodstream infections or unrelated concurrent joint infections.

### Ethics, funding, and potential conflicts of interest.

This study was approved by the Data Protection Officer (2018-105) at Akershus University Hospital. This study did not receive grants from public, commercial, or not-for-profit sectors. The authors report no conflict of interests.

## Results

Of the 123 patients included, 62 (50%) patients were female. The median age was 71 years (25–95).

### Time to microbiology results (Table 1)

Pathogens were identified after a median of 59 hours (2.5 days) and antibiotic recommendations were available after a median of 84 hours (3.5 days). Culturing results were finalized within a median of 141 hours (6 days).

**Table 1.** Time to results after conventional microbiologic diagnostics of patients with orthopedic implant-associated infections (n = 123)

Factor	Median (range)
Hours to pathogen identification	59 (16–238)
Hours to antibiotic recommendation	84 (36–238)
Hours to final results	141 (59–298)
Days from antibiotic recommendation to changed treatment (n = 66)	4 (0–24)

### Pathogens causing infections and culture-negative samples (Table 2)

Confirmed infection, defined by positive cultivation results, was observed in 109/123 (89%) patients. The remaining 14/123 (11%) patients had inconclusive/negative cultivation, of whom 4 patients had received antibiotic treatment prior to revision surgery. Monomicrobial infections were most common, identified in 79/109 (72%) patients. *S. aureus* and *S. epidermidis* were the most frequent pathogens. None of the *S. aureus* isolates and 10/18 of the *S. epidermidis* isolates were resistant to methicillin.

**Table 2.** Most common bacteria identified in orthopedic implant-associated infections

Identified bacteria	Patients n (%)	Number of patients with monomicrobial/ polymicrobial infection
*Staphylococcus aureus*	64 (52)	48/16
*Staphylococcus epidermidis*	18 (15)	7/11
*Cutibacterium acnes*	9 (7)	3/6
*Enterococcus faecalis*	11 (8)	2/9
Group B Я-hemolytic streptococci	8 (7)	5/3
*Staphylococcus capitis*	4 (3)	1/3
*Staphylococcus lugdunensis*	5 (4)	1/4
Others **^a^**	23 (19)	12/11
Culture negative/inconclusive	14 (11)	

**^a^** Pathogens found in 2 or fewer patients.

8 of 76 patients undergoing surgery during the microbiology lab’s opening hours had culture-negative biopsies, whereas 6 of 47 patients undergoing surgery outside opening hours had culture-negative biopsies. Similar results were seen in patients’ biopsies requiring pre-cultivation in broth (6/76 versus 3/47).

### Change of treatment

111/123 (90%) patients were given empirical treatment. Of the remaining 12/123 (10%) patients, 11 patients received targeted treatment based on previous infections and 1 patient did not receive any antibiotic treatment prior to cultivation results as infection was considered unlikely. Of the 109 patients with culture-positive results, antibiotic treatment was changed to targeted and narrowed treatment in 66 (61%) patients within a median of 4 days (0–24) based on the antibiotic treatment recommendations.

## Discussion

Our study confirms that conventional microbial diagnostics of OIAIs is comprehensive and time-consuming with a median of 2.5 days to pathogen identification and a median of 3.5 days to antibiotic recommendation. In addition, we identified a delay of median 4 days from when antibiotic recommendations were given to clinicians to when treatment was changed.

The lengthy time to results may be explained by a combination of several factors. The bacteria require time to multiply. In addition, 11% of the patients showed inconclusive or negative culturing, which involves 5 days of culturing before termination. Of the 109 patients with culture-positive results, 9 patients had positive samples only after pre-cultivation in broth. Pre-cultivation prolongs cultivation by 2 days. Lack of concurrence between the time of surgery and the opening hours of the microbiology lab may also prolong time to results. Biopsies taken after the lab’s opening hours were not cultivated until the following day in 47/123 patients. However, the concurrence between opening hours and time of surgery did not seem to affect the cultivation outcome. The frequency of inconclusive/negative results and positive results only after broth pre-cultivation did not differ among those patients with surgery performed before 16:00 compared with after 16:00.

Staphylococci are the most frequently reported causes of orthopedic implant infections (Arciola et al. 2018), as was confirmed by our study.

Of the 109 patients with culture-positive results, 66 received targeted treatment after receiving antibiotic resistance patterns. The majority of patients received better targeted antibiotic treatment, which may have led to more efficient treatment and reduced induction of antibiotic resistance. However, the response time from notification of antibiotic susceptibility results to the actual change of antibiotic treatment took a median of 4 days. This delayed response may negate the benefit of future rapid diagnostics. In our hospital, the microbiological results are sent electronically to the patient’s medical records immediately upon approval. Our continuous efforts to reduce the time to microbiological results will come to naught without clinicians reacting accordingly. Such optimization may also be relevant in diagnostics and treatment of other patient groups and types of infections.

As a retrospective study, this work is limited by the information already registered in the patients’ journals at the time of care. Furthermore, this study was carried out in a country with a low prevalence of antibiotic resistance, so empirical treatment success may not be comparable to countries with a higher antibiotic resistance load. The study’s strengths lie in the number of patients included, as OIAI is infrequent, and the patients coming from an unselected patient population.

As the majority of patients had culture-positive biopsies, more rapid diagnostics could improve time to targeted treatment and may potentially improve clinical outcome. Our study was not designed for patient-reported outcome measures (PROMS), but the obvious benefits would be faster diagnosis, and simpler, less resource-demanding care. An additional potential advantage is the reduction of antibiotic resistance development through more targeted and narrow-spectrum antibiotic treatment. This will require further investigation.

In conclusion, in taking 2.5 days for pathogen identification and 3.5 days for targeted treatment advice, conventional microbiological diagnostics of OIAI are not sufficiently expedient. Same-day diagnostics may contribute to rapid targeted treatment and more favorable clinical outcomes, but the delayed response from clinicians on the treatment recommendations needs to be addressed.

The authors would like to thank Tone Elin Langdal for help with extracting microbiological data.

Preliminary results were presented at the European Conference for Clinical Microbiology and Infectious Diseases (ECCMID) in Amsterdam, the Netherlands 2019 (Poster #2515).
